# The Knowledge and Application of Economics in Healthcare in a High-Income Country Today: The Case of Belgium

**DOI:** 10.3390/jmahp12030021

**Published:** 2024-09-04

**Authors:** Baudouin Standaert, Désirée Vandenberghe, Mark P. Connolly, Johan Hellings

**Affiliations:** 1 Department of Care & Ethics, Faculty of Medicine & Life Sciences, University of Hasselt, 3590 Diepenbeek, Belgium; desiree.vandenberghe@uhasselt.be (D.V.); johan.hellings@uhasselt.be (J.H.); 2 Global Market Access Solutions (GMAS), Charlotte, NC 28202, USA; mark@gmasoln.com; 3 Department of Pharmacoepidemiology and Pharmacoeconomics, Public University of Groningen, 9700 AB Groningen, The Netherlands

**Keywords:** economic assessment, healthcare, budgeting, financing, value assessment

## Abstract

Healthcare is a huge business sector in many countries, focusing on the social function of delivering quality health when people develop illness. The system is essentially financed by public funds based on the solidarity principle. With a large financial outlay, the sector must use economic evaluation methods to achieve better efficiency. The objective of our study was to evaluate and to understand how health economics is used today, taking Belgium as an example of a high-income country. The evaluation started with a historical view of healthcare development and ended with potential projections for its future. A literature review focused on country-specific evaluation reports to identify the health economic methods used, with a search for potential gaps. The first results indicated that Belgium in 2021 devoted 11% of its GDP, 17% of its total tax revenue, and 30% of the national Social Security Fund to health-related activities, totalizing EUR 55.5 billion spending. The main health economic method used was a cost-effectiveness analysis linked to budget impact, assigning reimbursable monetary values to new products becoming available. However, these evaluation methods only impacted at most 20% of the money circulating in healthcare. The remaining 80% was subject to financial regulations (70%) and budgeting (10%), which could use many other techniques of an economic analysis. The evaluation indicated two potentially important changes in health economic use in Belgium. One was an increased focus on budgeting with plans, time frames, and quantified treatment objectives on specific disease problems. Economic models with simulations are very supportive in those settings. The other was the application of constrained optimization methods, which may become the new standard of practice when switching from fee-for-service to pay-per-performance as promoted by value-based healthcare and value-based health management. This economic refocusing to a more constrained approach may help to keep the healthcare system sustainable and affordable in the face of the many future challenges including ageing, climate change, migration, pandemics, logistical limitations, and financial instability.

## 1. Introduction

Economists study and analyze individual and/or societal behaviour regarding the acquisition and use of limited resources for satisfying needs and wants (also called utilities) in pursuing personal and/or societal end goals [1]. Societal goals could be politically determined, encompassing diverse motivations such as group survival (prevalent in primitive societies), religious or spiritual beliefs, and the contemporary emphasis on welfare [2]. The framework for the realization of welfare is conceived and developed within welfare economics, focusing on production, consumption, and income increases [3,4]. Its paradigm incorporates a mixture of free-market economics, democratic principles, and governmental interventions for those constraints that are difficult to address by a purely free-market approach [5].

The field of economic study seeks to define methodologies for efficiently reaching economic objectives, and is characterized by optimizing output relative to limited input, given the context of diversity in age, sex, race, culture, education, demography, and/or geography [6,7]. Two of the best-known approaches to the economic study are free-market economics and planned economics [8]. The former is structured around the dynamics of a trade market, where supply and demand dictate the optimal price setting of goods and services, depending on their quantity and quality or their perceived value. A planned economy, in contrast, is not evidently dynamic, but is characterized by centralized control that determines what is produced, for whom, by whom, when, and at what price. Planned economics may also achieve societal welfare, but its long-term sustainability as a unique approach for trading may pose challenges [1].

Health economics (HE) emerged as a distinct field from general economics around six decades ago [9]. Its separate development was instigated due to the inadequacy of conventional economic principles in governing healthcare activities effectively. Issues like increasing costs, monopolistic price setting for products and services, and information imbalances between producers and users created distorted market conditions, resulting in disparate winners and losers [10,11]. Within the field of healthcare, modified situations of both free-market and planned economies coexist. They are intertwined, but they evolved independently over time [12]. HE within that context is not to be considered as a monolithic (one-method) application. It encompasses a very diverse approach to specific situations within healthcare [13]. The focus of the analysis presented here is on one country, Belgium, as an example for a high-income country. First, a synthesis of the historical development of healthcare is presented, seen from the perspective of payment allocations. It is important to understand the origin of the current healthcare financial situation. This is followed by a simplified structural presentation of the current healthcare organization for its economic assessment. Finally, different HE methods that should be considered are listed, based on publications in the literature that will help to identify the methods used in different situations. This triad package of basic information (history of payments, current organization of healthcare linked to economic assessment, and HE methods of evaluation) allows us to build an inventory of methods that are or could be applied across the healthcare system today to help with its challenges. The Discussion section considers possible future changes in HE, based on new evolutions expected in healthcare development.

## 2. Materials and Methods

### 2.1. Historical View

The starting point for healthcare development originated from the need to support individuals afflicted with illnesses needing professional medical intervention. This medical support was/is categorized as a social function, given that it is provided to individuals with no income due to their sickness and thereby requires remuneration from public resources for the professional assistance provided. The free-market economy, foundational to welfare development, was unable to equitably regulate the payment for the healthcare services delivered. Consequently, governmental intervention became necessary, leading to the establishment of State Social Security Funds based on the principle of solidarity [14]. This principle should ensure equitable access and remuneration for those in need of medical assistance, contingent upon prior contributions (premiums) made by those individuals as employees and/or employers [15]. The Results Section indicates how the money collected in this way is distributed across the different social support activities for which the government is responsible, based on actual data from Belgium.

### 2.2. Healthcare Organization for Application of Health Economics

**Figure 1:**
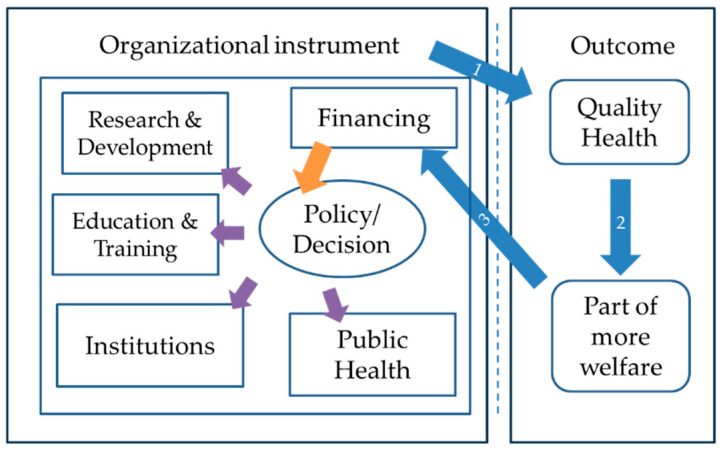
The organizational diagram of HC development in a country.

The organizational framework of healthcare as an economic activity can be illustrated through a simple diagram (Figure 1) [16]. As shown in Figure 1, a healthcare organization in a country should have a clear objective to produce quality health (arrow 1) as part of welfare (arrow 2). Having enough finances (arrow 3), the policy can make decisions directed to maximizing the efficiency and quality of the system, promoting equitable access, and sponsoring public health, training, and education, the institutions, and some research and development.

Economic understanding of this structure is to be stratified by economists into a well-known tripartite configuration, encompassing macro-, meso-, and micro-levels [12,17]. This stratification aims to facilitate a detailed understanding of the specific economic analyses that are required at each level, as described further in the Results Section.

Briefly, at the macro-level, healthcare considers a policy or strategic vision, characterized by the overarching financing mechanisms [18]. It also includes the activity of budgeting, which involves the identification of key healthcare challenges, developing a visionary plan, allocating budgetary provisions, and ongoing monitoring of quantified goals to be achieved within pre-specified timelines (Health System Performance Assessment [19]). At the meso (intermediate)-level, the focus shifts to the organization of care provision or supply [20,21]. This tier serves as the link between federal and regional policies with corresponding responsibilities in Belgium. At the micro-level, the intricacies of healthcare demand and its determinants, such as elasticity measurements, come under scrutiny [12]. This tier covers the determination of pricing for novel interventions, including medications, tests, diagnostics, devices, and vaccines [22]. Sometimes called the tactical level, this micro-level analysis is pivotal for comprehending the granular dynamics of healthcare economics and tailoring interventions accordingly.

### 2.3. Where and How Does HE Intervene?

The use of HE within healthcare extends beyond the conventional application of determining prices of medical goods entering the market [13,23]. The primary method employed here is a cost-effectiveness analysis (CEA) and budget impact analysis (BIA), to calculate the price of a new intervention based on health gain (i.e., its value) and the payer’s willingness to reimburse a unit of additional health gain [24,25,26]. The principle underlying this analysis is “more for more”: increased health gain is related to augmented financial input.

However, the economic issues affecting health and healthcare require much more diverse HE evaluation methods than just CEA and BIA to address distinct aspects of public healthcare resource allocation. Table 1 describes several recognized economic methods applicable to healthcare, categorized based on their focus on value measurement, on the one hand, being market-driven, or a planned economy, on the other hand, that can be split into budgeting considerations, or financing aspects [27]. The list is not exhaustive. The many books that are available on health economics have taken a larger perspective on the use and applications of different health economic methodologies in healthcare [28,29,30,31,32,33]. A specific focus on the three domains indicated in Table 1 has not often been observed in the literature but could be helpful, as explored in the Results Section.

To illustrate the applicability of the methods in Table 1, recently published examples of each method are presented in Appendix A. This should help to demonstrate when and how the different methods have been used. In the Results Section, the proportional distribution of the healthcare money flow in each of the different domains of market value measurement, and planned budgeting and financing, is analyzed for Belgium. The analysis also describes the HE methods commonly applied in each domain and indicates areas that are not yet applied in the system.

## 3. Results

### 3.1. Historical Overview

A concise historical overview of HE in Belgium reveals an evolution in parallel with the development of healthcare services, similar to many other high-income countries [34,35,36,37,38,39]. Total healthcare has evolved into a substantial economic sector for the country, not only by providing direct care but also through the production of healthcare resources (pharmaceuticals and devices) for the global market. Following the most recent data collection in the Eurostat Database of 2021, the sector constitutes 11% of the Gross Domestic Product, 17% of the tax revenue, and 30% of the Social Security Fund (EUR 55.5 billion total spent on reimbursed health-related activities), and engages 11% of the active working population in the country [40].

The funds collected by the State Social Security Fund are systematically distributed in Belgium by the National Social Security Office (NSSO) to diverse government departments. Presently, the most significant allocations are directed toward pensions, administered by the Federal Pension Services (FPS) with around 40% of the fund, and the National Institute for Sickness and Disability Insurance (NISDI) with around 30% of the fund for the reimbursement of healthcare services [41,42].

NISDI assumes the responsibility of redistributing the funds obtained from the NSSO, primarily through mutualities or insurance funds. These funds reimburse services performed by healthcare professionals based on predetermined reimbursement schemes agreed upon with the government. An exhaustive number of more than 15,000 distinct services are listed in the NISDI nomenclatura [43].

However, access to medical care in Belgium mandates membership in the recognized health insurance funds (see Appendix B). There are, legally defined, five such funds constituting the group of third-party payers. The concept, rooted in the ideas of Bismarck from Germany in 1875, significantly influences both the supply and demand of healthcare, shaping its evolution over a span of more than 70 years (from after World War II to present) [44]. As described by David Cutler of Harvard University in 2002, health system evolution is comprised of three phases [45]. The first phase focused on incorporating as many individuals as possible into the social insurance system through the mutual insurance companies, facilitating expansive healthcare coverage but lacking oversight on spending and service quality. The subsequent phase involved curtailing the excessive growth and spending. It resulted in resource constraints, prolonged waiting times, and suboptimal service delivery (1970 to 2000). The current period, from 2000 onward, constitutes the third phase, characterized by a pursuit of incentives seeking efficiency in service provision through the identification and active support of potential market forces.

Despite the absence of an explicit free-market scenario in healthcare—for instance, price setting and consumption are not set by free-market rules—the current economic activity of healthcare in Belgium involves a particular interplay between planned economy and market economy principles, such as free choice in the selection of medical care but within a fixed annual budget [46]. Moreover, the historical presence of inactive third-party payers with limited responsibility has the potential to induce financial imbalances, marked by excessive demand (negative moral hazard) and oversupply (supply-induced demand) [12]. However, the utilization of funds collected on the solidarity principle leans towards a planned economy split into financing (money transfer) and budget economy approaches (see Table 1). Consequently, contemporary economic assessment of healthcare in Belgium is perceived as a mix of planned and market economies, applied together but not optimally [41,47].

### 3.2. The Organization Levels

At the macro-level, financing results in a complex interplay of resource collection and redistribution, orchestrated through entities such as the NISDI and guided by a legal framework with annual consensus among stakeholders. A large part of the finance allocation (35–40%) is for hospital care. The latter has a complex structure of fixed payment per year through the Budget Monetary Fund (BMF) plus a flexible reimbursement depending on the activities performed by the professional healthcare physicians in the hospital unit. Annual evaluations about the financial performance of the hospitals expressed in expenses and profits are presented in the MAHA reports [48]. Economic assessments of comparative efficiency performance between hospital units have been developed using the technique of Data Envelopment Analysis (DEA) [49,50,51]. This type of evaluation is, however, new for Belgium. DEA is helpful in evaluating whether important deviations in efficiency are observed between hospital units, caused by scale or technical changes in the individual organizations.

Meanwhile, there is currently an interesting change in emphasis within the domain of a planned economy towards budgeting instead of financial or money transfer. Currently, the budgeting approaches in the planned economy cover only a marginal part of the total money circulation in healthcare (see Table 2). Appendix C lists the activities to be performed under this budgetary framework as an example. The identification of those critical issues is often informed by comparative research at a national or regional level that is heavily encouraged by the European Commission, and also by the Organisation for Economic Co-operation and Development (OECD), playing a critical role in country comparison within market-driven organizations [19,52,53].

At the meso-level, the Flemish region endeavours to formulate strategic hospital plans within networks, aligning with federal directives to optimize hospital bed utilization in their network plans [54]. The meso-level becomes significant as a point of collaborative consultation, facilitating the joint development of essential healthcare services that align with financial considerations.

The micro-level exhibits a pronounced federal responsibility. However, notable asymmetry exists in the federal relationship with the regions concerning pricing mechanisms of specific products and services, such as vaccines and prevention programmes.

Analyzing the monetary flows within the healthcare landscape of Belgium reveals distinct economic approaches, as presented in Table 2. Predominantly financial transactions account for a very substantial proportion (70%), while value-based pricing constitutes a comparatively much smaller share (20%) of the monetary flows [55]. The allocation of funds towards budgeting activities has historically been limited, gaining prominence only recently, commencing in the year 2021 and currently accounting for 10% of monetary flows [56].

The proposed division of the healthcare system into the three economic levels (macro, meso, micro) enables a more nuanced understanding of the role of HE and its corresponding interventions at each level. Table 3 describes specific HE contributions to each tier, highlighting its overarching objective of facilitating more responsible (equitable) and efficient (cost, technical, or allocative) management within the system. However, the ultimate goal of using HE is to maximize the delivery of services towards quality health outcomes while operating within specific constraints, such as available resources, logistics, personnel, and equality of access.

At the macro-level, the effectiveness of the healthcare system is shaped by economic analyses that define the annual co-determination of the cost growth rate, social contributions collected for the NSSO, and out-of-pocket (OOP) contributions or co-payments. Conversely, the health system can shape the meso- and macro-economy through changes in population health resulting in productivity gains (fiscal health modelling (FHM)). These economic interventions are aligned with the characteristics of a financially focused economy, overall and specifically. The economic evaluations play a pivotal role in setting limits on changes in supply (offer) and demand (market economy) by assessing the appropriateness of social contributions and the OOP levels. Budgeting, an essential, newly grounded development in comparative data examination (evidence-based), is gaining prominence. Planned economy approaches therefore offer at the level of finance and budgeting the potential for better control of cost and outcome in specific healthcare challenges.

At the meso-level, HE engages in the study of all supply data as a function of subregional epidemiological analyses (population health and management) [57]. It can help with defining the number of beds needed for hospital care, nursing homes, and mental care. It can facilitate an understanding of local needs and demands for diverse provisions of elderly care and mental healthcare. Economic evaluations at this level aid in determining the most effective financing mechanisms for interventions in both the short and long term, and in the development of population health management [57]. Additionally, HE encourages regulated market competitiveness among the five mutuality funds in Belgium, which may aim to enhance customer loyalty by proposing superior care on advantageous terms, thereby seeking better services at competitive market prices [58].

The micro-level focuses on understanding individual demand and the corresponding supply responses. Key aspects include evaluating the impact of changes in co-payments, income levels, and life expectancy on supply and demand dynamics, expressed through the elasticity ratios of relative changes. HE, as mentioned earlier, is notable for its application of CEA and BIA in pricing new products entering the market [24,32]. This market-based approach aims to ascertain the utilities generated by a new medical product, measured in terms of individual clinical added value, such as the Quality Adjusted Life Year (QALY) for which society is willing to pay a maximum price per unit gained. The micro-level also assesses the economic added value of innovation and prevention over treatment.

In summary, most of the healthcare budget in Belgium, approximately 80%, could be evaluated under the economic guidance of financial and budget planned economic rules. The remaining 20% is assessed using value assessment methods, including CEA and BIA. This distribution of money circulation in the three domains of value assessment, budgeting, and financing indicates that training and education in HE currently has too great a focus on value and places much less emphasis on financing and budgeting [59].

### 3.3. Challenges to the Sustainability of Healthcare

**Figure 2:**
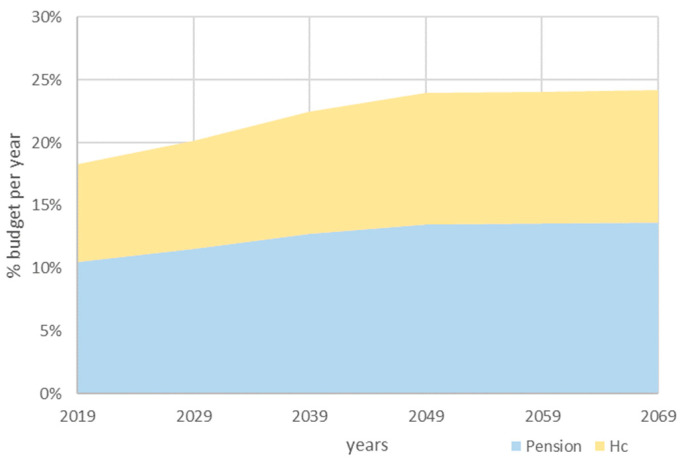
The estimated budget increase in the social support functions of pensions and HC in Belgium in the future (2019–2070).

Examining the evolution of healthcare costs over the past decades reveals a pattern of annual increases occasionally punctuated by spikes, such as during the COVID-19 pandemic [60]. This pattern with fluctuations raises concerns about the future viability and affordability of the healthcare system. The factors contributing to the annual increases are well-known and include increased demand, elevated expectations, an ageing population requiring increased healthcare interventions, and new, more expensive medical technologies for laboratory tests, diagnostics, and pharmaceuticals. The challenge lies in organizing a financial framework capable of managing growth, while ensuring the system’s long-term sustainability and durability [61]. Figure 2 illustrates the projected budgetary escalation of two social support functions (pensions and healthcare) in Belgium from 2019 to 2070, offering a visual depiction of the anticipated challenges in sustaining the healthcare system, particularly if the diminishing workforce still remains the primary source of funding [62].

**Figure 3:**
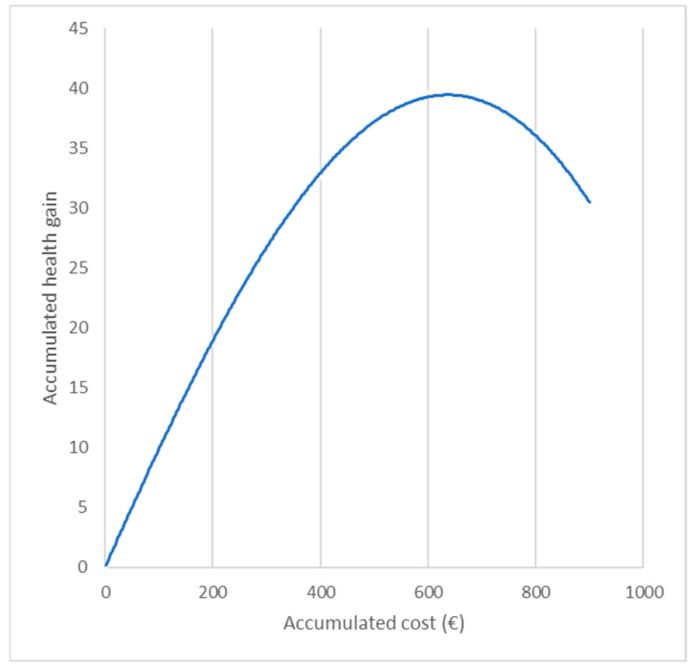
The productivity curve between health gain and investment [63].

In managing these challenges, the application of HE emerges as a key tool to inform decision-making. An example is presented in Figure 3, which demonstrates the dynamic relationship between increased investment in healthcare and the resultant health gains [63]. The simulated production function exhibits a non-linear trajectory, indicating that increased investments lead to extended life expectancy, but the associated health gain reaches a peak and then declines as more aged individuals become exposed to age-related diseases that currently lack appropriate treatment, such as cancer and dementia. The graph highlights important areas for future research, such as addressing complex disease burdens in elderly populations.

The challenges confronting the healthcare system extend beyond this financial constraint. Climate change (manifesting as extreme weather conditions), new pandemics, financial instability, migration, and enhanced mental healthcare potentially pose high new hurdles [64]. Moreover, these challenges are interconnected, forming links where the activation of one may increase the impact of others, necessitating an integrated approach to address the multifaceted issues.

## 4. Discussion

The underutilization of the many HE methods available may be attributed to the absence of awareness, education, and training, limiting their potential to enrich efficiency in overall healthcare management. However, a tremendous improvement has occurred at the federal level in Belgium since the Knowledge Centre of Excellence (KCE) institute was installed and became operational in 2003 [65]. This paper has described the traditional role of HE and its economic interventions, characterized as a sort of cosmetic alteration to the healthcare system. The interventions, such as budgeting and evidence-based implementation protocols, have had limited impact on the fundamental working habits of healthcare professionals. The challenge lies in the fact that these economic measures do not directly influence the core operations of healthcare delivery or address the efficiency of care and its quality from the patient’s perspective.

**Figure 4:**
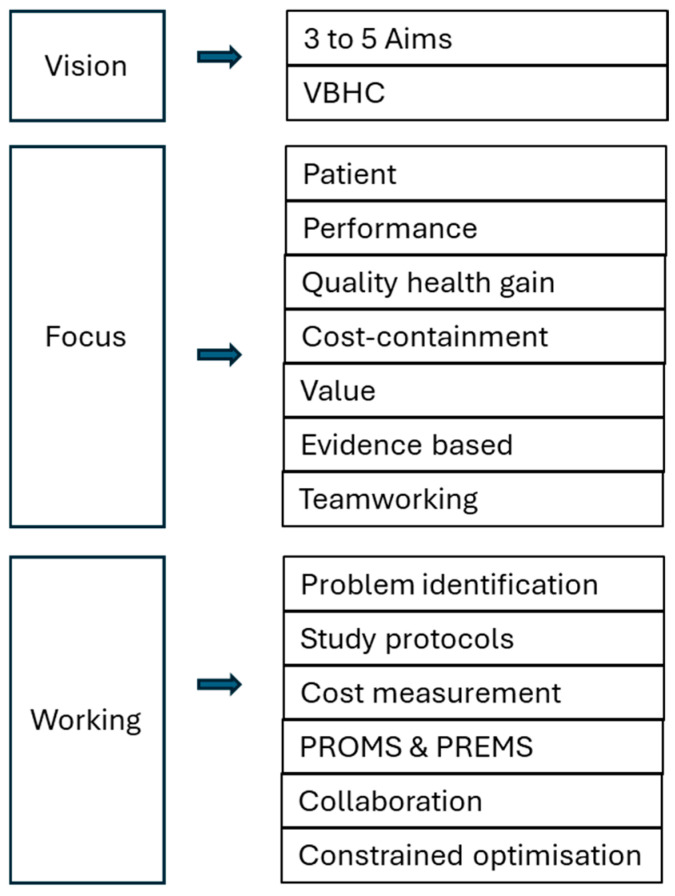
Refocusing HC development.

This realization suggests a re-evaluation of the approach to healthcare, necessitating a shift in focus from input-based evaluations (counting services provided) to output-oriented assessments (performance outcomes) (see Figure 4). The historical lack of precise evaluations of care performance has led to an inherent limitation in achieving effective cost control. In attempting to break through this limitation, a change in thinking emerged from the United States at the beginning of this century, promoting a vision of carrying out what is right and demanded by the patient, at a reasonable price [66]. This revolutionary approach to healthcare has been spearheaded by the Institute of Health Innovation (IHI) in the US, promoting first the Triple followed by the Quadruple and Quintuple Aim, and by Michael Porter’s vision of value-based healthcare (VBHC) [67,68,69,70]. The crux of this paradigm shift lies in refocusing on the patient in care, assessing the outcomes of interventions, evaluating the value of work performed within budget constraints, promoting teamwork, and adhering to evidence-based treatment pathways.

In pursuit of this transformative approach, economic evaluation must evolve beyond the traditional CEA model to embrace constrained optimization (CO). CO entails determining the forms of treatment or combinations of treatments that maximize health gains from the patient’s perspective, considering specific constraints, with a fixed budget or a known budget increase. The DEA method used to evaluate the efficiency of hospital units is in that respect a concrete application of CO. The paradigm shift towards CO is encapsulated in the new economic assessment required for VBHC. While CEA may still be relevant for defining the price of new products, VBHC demands a distinct economic evaluation focusing on optimizing patient care within existing budgetary constraints, irrespective of potential budget increases [71].

The evolution of the Belgian healthcare system over the past seven decades has been remarkable, evolving into a major economic entity sustained by contributions from individuals through earnings and taxes. This system, intricately present in Belgian societal functioning, has historically experienced increasing financial allocations. It is only in the past 15 years that a profound reality has surfaced: the need to evaluate the healthcare system not merely on the inventory of services accomplished, but on the outcomes delivered for individuals and society [71,72].

Remarkably, economic scrutiny in healthcare to date, with CEA methods designed over the past four decades for assessing new interventions, has typically focused on a fraction of the monetary spending on healthcare, approximately 20%. A shift in attention is needed, directed towards enhancing efficiency in the monetary utilization of the remaining 80%, with the use of methods including budget and financial evaluations (see Table 1). This entails a multifaceted approach encompassing cost, technical, and allocative efficiencies, with a renewed emphasis on preventive measures over curative interventions. This indicates the exploration and exploitation of an economy of budgeting, a domain worthy of further investigation.

Moving forward, value-based health management (VBHM) emerges as a compelling incentive, away from the constricting confines of VBHC, which centres too much around care and cure [73]. The shift towards a more inclusive vision, integrating prevention, care, and cure, aligns with the imperative to maintain health support (prevention + care + cure) as an affordable, sustainable, and enduring societal framework. It underscores a holistic approach to HE, with an unwavering commitment to securing the well-being of individuals and the broader community.

What is presented here is not completely new. It is a way of looking at different blocks present in the healthcare system and focusing on specific aspects of the way money circulates in the healthcare system that could be based on value, budget, or finance [74,75]. Each block needs to understand and accept that there are other ways of circulating and appreciating money in the system to obtain durability, sustainability, affordability, and quality care. Working together should help to support longer and healthier survival of people and the healthcare organization.

## Figures and Tables

**Table 1:** Methods of economic evaluation applicable to HC.

Market	Planned	
Value	Budget	Finance
Incremental CEA	BIA	FHM cohort
Decremental CEA	Cost minimisation	FHM-SAM
Distributional CEA	CBA	Poverty trap avoidance
Extended CEA	CO	RSA
MCDA/SMAA	Portfolio management	Macro-economics
Cost–consequence analysis	Cobb–Douglas function	Production functions
Cost–impact analysis	ROI and SROI	
Tiered and volume pricing	DEA	

BIA: budget impact analysis; CBA: cost–benefit analysis; CEA: cost-effectiveness analysis; CO: constrained optimization; FHM: fiscal health modelling; MCDA: multicriteria decision-making analysis; ROI: return on investment; RSA: risk sharing agreement; SAM: social accounting matrix; SMAA: stochastic multicriteria acceptability analysis; SROI: social return on investment; DEA: data envelopment analysis.

**Table 2:** Economic handling in HC in Belgium.

Overall	Planned-Driven		Market-Driven
What?	Finance	Budget	Value
Definition	Reimbursable and registered medical activities	Action plan, time schedule, goals, budget scheme	Value-based pricing
How much?	70%	10%	20%
Methods?	Fiscal health modelling Macro-economics	Budget impact + constrained optimization Cobb–Douglas function DEA	Cost-effectiveness analysis Cost–impact analysis
Influencer?	Hospital network	Europe—OECD	PPF to PPP
Logic?	Constrained budget		More for more
Who?	NSSO insurance payers		Producers

DEA, Data Envelop Analysis; NSSO, National Social Security Office; OECD, Organisation for Economic Co-operation and Development; PPF: Pay Per Fee; PPP: Pay Per Performance.

**Table 3:** Current and potential economic activities in HC organizations at the three levels of macro, meso, and micro.

Level	Application	Activity	Type of Economic Approach	Method
Macro or Strategic	Financial	Defining the annual growth rate; nomenclatura; BFM	Financial economy	FHM; macro-economy; production function
	Contribution	OOP + social	Financial + market economy	FHM; SAM
	Budgeting	Plan, scheme, target (quantified)	Budget economy	CO; portfolio; Cobb–Douglas; DEA
Meso or Offer/Supply	Network Regions	Hospitalisations; health subregions; population health	Budget economy	Cost–consequence; DEA; CO
	Offer Limitations	Grants/regional taxations	Financial economy	Modeling + simulations
	Mutualities/Insurance	Regulated competition	Market economy	FHM
Micro or Tactical	Demand/Offer	Elasticities	Market economy	Budget/outcome; CO
	HTA	Pricing	Market economy	CEA-BIA; CBA; RSA; CIA; FHM

HTA: Health Technology Assessment; BFM: Budget van de Financiële Middelen (FRB); OOP: Out of Pocket; FHM: Fiscal Health Modelling; SAM: Social Accounting Matrix; CO: Constrained Optimization; CEA: Cost-Effectiveness Analysis; BIA: Budget Impact Analysis; CBA: Cost–Benefit Analysis; RSA: Risk Sharing Agreement; CIA: Cost–Impact Analysis; DEA: Data Envelop Analysis.

## Data Availability

All data presented in the document come from publicly available data sources like Eurostat, NSSO, NISDI.

## Appendix A

List of projects and publications following HE evaluations with a different focus on value, budget, or finance:

**Value Assessment**

*Incremental cost-effectiveness analysis* [76,77]

*Decremental cost-effectiveness analysis* [78,79]

*Distributional cost-effectiveness analysis* [80,81]

*Extended cost-effectiveness analysis* [82,83]

*MCDA/SMAA* [84,85,86]

*Cost–consequence analysis* [87,88]

*Cost–impact analysis* [89,90]

*Tiered pricing* [91]

**Budget Assessment**

*Budget impact analysis* [92,93]

*Cost minimisation* [94,95]

*Cost–benefit analysis* [96,97]

*Constrained optimization* [98,99,100]

*Portfolio management* [101,102]

*Cobb–Douglas function* [103,104]

*Return on Investment (ROI)* [105,106]

*DEA* [49,107]

**Finance Assessment**

*Fiscal health modelling cohort* [108,109]

*Fiscal health modelling—Social Account Matrix (SAM)* [110,111]

*Poverty trap avoidance* [112,113,114]

*Risk sharing agreement (RSA)* [115,116,117,118]

*Macro-Economics* [119,120,121]

*Production function* [122]

## Appendix B

### Mutualities and Other Third-Party Payers in Belgium

In Belgium, health insurance is needed to obtain access to the professional medical services. It costs around EUR 10 per month. Being insured allows for visits to a general practitioner (GP) and hospital, and obtaining prescribed medicine. Health insurance requires joining an insurance institution, by completing a registration form from the institution selected, which may be the Auxiliary Health and Disability Insurance Fund (HZIV) or one of the following five health insurance funds:

- Christian mutuality;
- Neutral mutuality health insurance fund;
- Socialist mutualities (Bond Moyson, De Voorzorg);
- Liberal mutuality health insurance fund;
- Independent health insurance funds (Onafhankelijk Ziekenfonds OZ, Securex, Partena, Helan).

A mutuality health insurance fund is not legally obliged to register everyone (risk selection is therefore possible), but the Auxiliary Fund can never refuse a registration. The Auxiliary Fund does not require payment of a membership fee.

## Appendix C

Seven transversal projects (https://www.riziv.fgov.be/nl/thema-s/financiering-van-de-verzekering/budget-van-de-ziekteverzekering-meerjarig-en-dynamisch-vanaf-2022 (accessed on 20 February 2024)):

1. A preliminary care pathway for patients at risk of diabetes and follow-up of patients with diabetes;
2. A childhood obesity care pathway;
3. A multidisciplinary perinatal (prenatal and postnatal) care pathway for vulnerable women;
4. A care pathway around the patient pre- and post-abdominal organ transplantation;
5. Investing in psychiatric care with special focus on somatic care and young people with psychiatric problems;
6. Reducing readmissions (new hospitalization periods) by rolling out better pulmonary rehabilitation and increasing the quality of life of the patients concerned by improving their functional capabilities;
7. Various secondary and tertiary prevention first-line projects, including post-COVID-19 ones.
